# Remaining missed opportunities of child survival in Peru: modelling mortality impact of universal and equitable coverage of proven interventions

**DOI:** 10.1186/s12889-016-3668-7

**Published:** 2016-10-04

**Authors:** Yvonne Tam, Luis Huicho, Carlos A. Huayanay-Espinoza, María Clara Restrepo-Méndez

**Affiliations:** 1 Institute for International Programs and Department of International Health, Bloomberg School of Public Health, Johns Hopkins University, 615 N. Wolfe Street, Baltimore, MD 21205 USA; 2 Centro de Investigación para el Desarrollo Integral y Sostenible, Universidad Peruana Cayetano Heredia, Lima, Peru; 3 Centro de Investigación en Salud Materna e Infantil, Universidad Peruana Cayetano Heredia, Lima, Peru; 4 School of Medicine, Universidad Peruana Cayetano Heredia, Lima, Peru; 5 Universidad Nacional Mayor de San Marcos, Lima, Peru; 6 International Center for Equity in Health, Federal University of Pelotas, Pelotas, Brazil

## Abstract

**Background:**

Peru has made great improvements in reducing stunting and child mortality in the past decade, and has reached the Millennium Development Goals 1 and 4. The remaining challenges or missed opportunities for child survival needs to be identified and quantified, in order to guide the next steps to further improve child survival in Peru.

**Methods:**

We used the Lives Saved Tool (LiST) to project the mortality impact of proven interventions reaching every women and child in need, and the mortality impact of eliminating inequalities in coverage distribution between wealth quintiles and urban–rural residence.

**Results:**

Our analyses quantified the remaining missed opportunities in Peru, where prioritizing scale-up of facility-based case management for all small and sick babies will be most effective in mortality reduction, compared to other evidenced-based interventions that prevent maternal and child deaths. Eliminating coverage disparities between the poorest quintiles and the richest will reduce under-five and neonatal mortality by 22.0 and 40.6 %, while eliminating coverage disparities between those living in rural and urban areas will reduce under-five and neonatal mortality by 29.3 and 45.2 %. This projected neonatal mortality reduction achieved by eliminating coverage disparities is almost comparable to that already achieved by Peru over the past decade.

**Conclusions:**

Although Peru has made great strides in improving child survival, further improvement in child health, especially in newborn health can be achieved if there is universal and equitable coverage of proven, quality health facility-based interventions. The magnitude of reduction in mortality will be similar to what has been achieved in the past decade. Strengthening health system to identify, understand, and direct resources to the poor and rural areas will ensure that Peru achieve the Sustainable Development Goals by 2030.

**Electronic supplementary material:**

The online version of this article (doi:10.1186/s12889-016-3668-7) contains supplementary material, which is available to authorized users.

## Background

In the past decade, coverages of child health interventions in Peru have greatly improved, and have led to significant reduction in stunting and child mortality. Among the 75 low and middle income countries that contribute to the majority of child deaths in the world, Peru had the second highest annual rate of reduction (6.2 %) in under-five mortality rate, achieving a 58 % reduction in under-five mortality rate between 2000 and 2013 [1]. Peru is one of the few countries that have achieved the UN Millennium Development Goals (MDGs) 1 and 4, having reduced the number of underweight children by half, and the under-five child mortality by two-third between 1990 and 2015 [2]. Peru has also made significant progress in reducing socioeconomic and urban–rural inequalities in coverage, mortality, and stunting prevalence. These improvements were credited to a combination of political will, economic growth, improvement in social determinants of health, and sustained implementation of proven high-impact interventions via a pro-poor approach [1].

Although Peru has made great improvements in public health, remaining challenges include strengthening health sector development to achieve universal health coverage, and addressing the remaining inequalities in wealth distribution, poverty, and access to health services [1]. Prioritizing scale-up of proven interventions that can save the most lives can further help to reduce mortality. It is also important to understand who are the remaining population segments that need these life-saving interventions for child survival, but are not receiving them.

This paper sets out to use the Lives Saved Tool (LiST) to assess the impact on child mortality when proven health interventions reach every women and child, and the impact of removing coverage inequality such that coverage of health interventions is the same regardless of household wealth or location. If there is no universal and equitable access to these effective interventions, the potential lives saved become ‘missed opportunities’.

## Methods

We used the Lives Saved Tool (LiST) to model scenarios to quantify the missed opportunities in Peru. LiST is a modeling software that projects mortality impact from changes in coverage of maternal, neonatal, and child health interventions. Coverage is defined as the proportion of population that received the health intervention they need. LiST is a deterministic model that characterizes the fixed, mathematical relationships between inputs and outputs; the same inputs will produce the same outputs. Further methods and assumptions used in LiST have been previously described [3]. LiST contains published country-level input estimates such as population size, total fertility rate, mortality rate, disease burden distribution, and intervention coverage that users can use or customize to establish a baseline for modelling. Impacts of interventions are linked to one or more cause-specific deaths based on published evidence of efficacy. Published efficacy values are generated according to rules of the Child Health Epidemiology Reference Group (CHERG) [4]. When users put in a change of intervention coverage, the model calculates the impact on mortality, and re-project input estimates that were used in the baseline for future years. LiST modeling does not separately compute the effects of distal factors that impact child survival, such as social determinants of health or out-of-health sector changes on mortality and nutrition. Any improvements in these distal factors are assumed to have increased interventions’ coverage, which would be reflected in the coverage estimates from large household surveys used as inputs for LiST modeling [5]. LiST is housed within Spectrum - a suite of several modelling software for decision making in program planning and policy making, and is available for the public to download for free [6]. More information about LiST can be found on livessavedtool.org. Results shown in this paper were generated using Spectrum version 5.32, downloaded July, 2015 (http://www.avenirhealth.org/software-spectrum.php).

Baseline scenario for national Peru and rural Peru are created using the most recent coverage estimates from DHS 2012 [7, 8], and are described in the Additional file 1: Tables S1 and S3. Sources for other baseline inputs for national Peru and rural Peru are described in the Additional file 1: Tables S2 and S4, and Figure S1 and S2 [9–11]. Where published baseline inputs for rural Peru are not readily available, for example disease burden distribution and mortality rates, we used LiST to project that for rural Peru based on coverage difference between national Peru and rural Peru [12]. We also assumed that the trend of change for population structure and total fertility rate over time of rural Peru will be the same as national Peru.

Three sets of scenarios were built to look at the impact of interventions reaching universal coverage, and the impact of eliminating inequitable distribution of health intervention coverage in Peru, by household wealth and location. Please refer to the additional file for input estimates used.

1. Impact of individual interventions reaching universal coverage in national Peru:
  In this scenario, we looked at the individual impact of each intervention if it reaches universal coverage, while coverage of all other interventions stay constant at its current coverage until the next year. Taking into account the current disease burden distribution and efficacy of interventions, each intervention is scaled up from its current coverage in 2012 to 90 % in 2013. Ninety percent is chosen as the target universal coverage as it is an aspirational but achievable target, as evidenced by the coverage achieved by DPT3 vaccination in many low and middle income countries [13]. If current coverage of intervention is at or higher than 90 %, the intervention is not scaled down and not included in this scenario.
2. Impact of removing inequitable distribution of coverage due to household wealth
  In this scenario, we looked at the impact if coverage of the national population is scaled up to coverage of the richest quintile in the next year. This scenario is designed to capture the impact if coverages of population in the poorer quintiles are brought up to the level of the richest quintile.
3. Impact of removing inequitable distribution of coverage due to household location
  In this scenario, we looked at the impact if coverage of the rural population is scaled up to coverage of the urban population in the next year.
  Main outcomes of interest of these three scenarios include lives saved (or additional deaths prevented) and mortality rate reduction for neonates (i.e. children under 1 month) and children under-five. These outcomes are aggregated impact from 2013 to 2017, because vaccines and other interventions that reduce stunting and wasting have impacts over the years as a birth cohort ages out.

## Results

1. Impact of individual interventions reaching universal coverage in national Peru (Scenario “Universal coverage” in figures):
  Figure 1 shows the aggregated impact for each intervention for years 2013 to 2017 if their current coverage in 2012 were brought up to universal coverage in 2013. Interventions such as full supportive care for prematurity and labor and delivery management in the Comprehensive Emergency Obstetric Care (CEmOC) level each prevent about 900 deaths. Most of these are neonatal deaths prevented, and each of these interventions prevents more than double the number of deaths prevented for other interventions modelled. Interventions’ impacts were also ranked according to the population group it affects. For pregnant women, the most impactful intervention is maternal sepsis case management, preventing 108 maternal deaths. For stillbirths, labor and delivery management in the CEmOC level is the most impactful, preventing 250 stillbirths. For neonates, full supportive care for prematurity prevents 901 neonatal deaths. As for children 1 to 59 months, the missed opportunities are in curative interventions. A universal coverage of therapeutic feeding for children that are severely stunted will prevent 301 child deaths. Please refer to Figures S3–S6 in the Additional file 1 for interventions’ impact ranked by population groups.

**Fig. 1:**
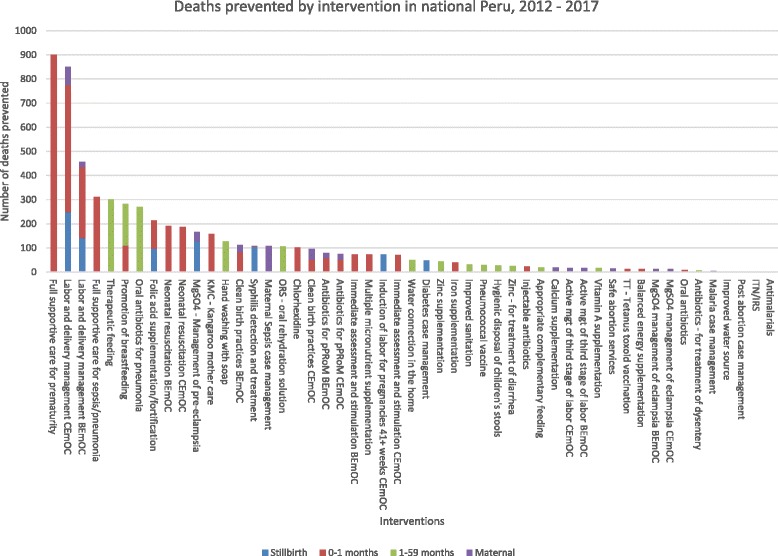
Total deaths prevented by intervention in national Peru, 2012 to 2017 for scenario “Universal coverage”
2. Impact of removing inequitable distribution of coverage due to wealth of household (Scenario “Peru national to richest” in tables)
  Majority of the interventions’ coverage estimates of the richest are higher than that of the poorer quintiles. A decrease in coverage of intervention will result in negative deaths prevented.
  If coverage of the poorer quintiles were brought up to match coverage of the richest quintile, about 12,000 child deaths will be prevented, resulting in a 22.9 % reduction in child deaths in 2017 relative to 2012. Under-five mortality rate drops from 19.1 to 14.9 (22.0 % reduction) (Tables 1 and 2). Of the children under-five, about 10,900 neonatal deaths will be prevented, resulting in a 41.5 % reduction in neonatal deaths in 2017 relative to 2012. Neonatal mortality rate drops from 9.4 to 5.6 (40.6 % reduction) (Tables 2 and 3).

**Table 1:** Coverage change, under-five deaths prevented and under-five mortality reductions by intervention for scenario “Peru national to richest”

Peru national to richest			
Intervention	Coverage change from national Peru to richest Peru (%)	Additional deaths prevented in children under-five by intervention	Percent of under-five mortality reduction attributable to intervention (%)
Full supportive care for prematurity	35.0	5,805	48.5 %
Full supportive care for sepsis/pneumonia	35.0	2,503	20.9 %
Labor and delivery management	11.1	2,501	20.9 %
Reduction of stunting	−11.3	1,443	12.1 %
Water connection in the home	17.9	641	5.4 %
Improved sanitation - Utilization of latrines or toilets	37.8	394	3.3 %
*H. influenzae b* vaccine	4.9	375	3.1 %
Neonatal resuscitation	13.8	361	3.0 %
Reduction of wasting	−0.1	287	2.4 %
Antibiotics for pPRoM^a^	35.0	270	2.3 %
Clean birth practices	11.1	145	1.2 %
Immediate assessment and stimulation	11.1	105	0.9 %
Clean postnatal practices	4.6	90	0.8 %
Oral antibiotics for pneumonia	1.2	85	0.7 %
DPT vaccine	4.9	76	0.6 %
ORS - oral rehydration solution	1.9	30	0.3 %
Syphilis detection and treatment	23.7	15	0.1 %
Vitamin A supplementation	−3.5	−5	0.0 %
Improved water source	13.2	−20	−0.2 %
Hygienic disposal of children’s stools	−5.4	−25	−0.2 %
Thermal care	−21.2	−454	−3.8 %
Injectable antibiotics	−21.2	−668	−5.6 %
Promotion of breastfeeding	−17.8	−1,986	−16.6 %
Total		11,968	100

^a^ *pPRoM* preterm premature rupture of membranes

**Table 2:** Under-five deaths and mortality rates 2012 to 2017 for scenario “Peru national to richest”

Peru national to richest	2012	2013	2014	2015	2016	2017	% reduction 2017 relative to 2012
Deaths in children under five years of age							
Total (0–59 months)	11,031	8,651	8,583	8,554	8,536	8,502	−22.9
< 1 month	5,435	3,211	3,203	3,195	3,187	3,178	−41.5
1-59 months	5,595	5,440	5,380	5,358	5,349	5,324	−4.8
Mortality rates summary							
Neonatal mortality rate (deaths per 1,000 live births)	9.4	5.6	5.6	5.6	5.6	5.6	−40.6
Under five mortality rate (deaths per 1,000 live births)	19.1	15.0	14.9	14.9	14.9	14.9	−22.0

**Table 3:** Coverage change, neonatal deaths prevented and neonatal mortality reduction by intervention for scenario “Peru national to richest”

Peru national to richest			
Intervention	Coverage change from national Peru to richest Peru (%)	Additional deaths prevented in neonates by intervention	Percent of neonatal mortality reduction attributable to intervention (%)
Full supportive care for prematurity	35.0	5,805	53.0
Full supportive care for sepsis/pneumonia	35.0	2,503	22.9
Labor and delivery management	11.1	2,501	22.8
Neonatal resuscitation	13.8	361	3.3
Promotion of breastfeeding	−17.8	275	2.5
Antibiotics for pPRoM^a^	35.0	270	2.5
Clean birth practices	11.1	145	1.3
Immediate assessment and stimulation	11.1	105	1.0
Clean postnatal practices	4.6	90	0.8
Syphilis detection and treatment	23.7	15	0.1
Thermal care	−21.2	−454	−4.1
Injectable antibiotics	−21.2	−668	−6.1
Total		10,948	100.0

^a^ *pPRoM* preterm premature rupture of membranes
3. Impact of removing inequitable distribution of coverage due to location of household (Scenario “Peru rural to urban” in tables)
  Majority of the interventions’ coverage estimates of the urban population are higher than that of the rural population.
  If coverage of the rural population were brought up to match coverage of the urban population, about 6,300 child deaths will be prevented, resulting in a 26.2 % reduction in child deaths in 2017 relative to 2012. Under-five mortality rate drops from 22.3 to 15.7 (29.3 % reduction) (Tables 4 and 5). Of the children under-five, about 5,000 neonatal deaths will be prevented, resulting in a 42.9 % reduction in neonatal deaths in 2017 relative to 2012. Neonatal mortality rate drops from 11.1 to 6.1 (45.2 % reduction) (Tables 5 and 6).

**Table 4:** Coverage change, under-five deaths prevented and under-five mortality reduction by intervention for scenario “Peru rural to urban”

Peru rural to urban			
Intervention	Coverage increase from Peru rural to Peru urban (%)	Additional deaths prevented in children under-five by intervention	Percent of under-five mortality reduction attributable to intervention (%)
Full supportive care for prematurity	46.2	2,676	42.1
Full supportive care for sepsis/pneumonia	46.2	1,336	21.0
Labor and delivery management	23.8	1,215	19.1
Reduction of stunting	−14.6	1,001	16.6
Water connection in the home	25.3	347	5.8
Neonatal resuscitation	29.9	332	5.2
Improved sanitation - Utilization of latrines or toilets	46.1	206	3.4
Antibiotics for pPRoM^a^	46.2	162	2.5
Clean birth practices	23.8	147	2.3
Immediate assessment and stimulation	23.8	100	1.6
Clean postnatal practices	9.8	93	1.5
ORS - oral rehydration solution	12.0	77	1.3
Reduction of wasting	−0.17	60	1.0
Oral antibiotics for pneumonia	2.0	43	0.7
*H. influenzae* b vaccine	0.8	24	0.4
DPT vaccine	1.4	10	0.2
Syphilis detection and treatment	23.3	5	0.1
Hygienic disposal of children’s stools	2.1	5	0.1
Vitamin A supplementation	−5.9	−5	−0.1
Improved water source	21.5	−9	−0.1
Thermal care	−16.3	−157	−2.5
Injectable antibiotics	−16.3	−259	−4.1
Promotion of breastfeeding	−18.4	−1,108	−17.7
Total		6,301	100.0

^a^ *pPRoM* preterm premature rupture of membranes

**Table 5:** Under-five deaths and mortality rates 2012 to 2017 for scenario “Peru rural to urban”

Peru rural to urban	2012	2013	2014	2015	2016	2017	% reduction 2017 relative to 2012
Deaths in children under-five years of age							
Total (0–59 months)	4,251	2,594	3,062	3,088	3,116	3,138	−26.2
< 1 month	2,145	1,185	1,193	1,204	1,214	1,224	−42.9
1–59 months	2,106	1,409	1,869	1,884	1,901	1,914	−9.1
Mortality rates summary							
Neonatal mortality rate (deaths per 1,000 live births)	11.1	6.1	6.1	6.1	6.1	6.1	−45.2
Under-five mortality rate (deaths per 1,000 live births)	22.3	15.8	15.8	15.8	15.8	15.7	−29.3

**Table 6:** Coverage change, neonatal deaths prevented and neonatal mortality reduction by intervention for scenario “Peru rural to urban”

Peru rural to urban			
Intervention	Coverage Increase from Peru rural to Peru urban (%)	Additional deaths prevented in neonates by intervention	Percent of neonatal mortality reduction attributable to intervention (%)
Full supportive care for prematurity	46.2	2,676	116.8
Full supportive care for sepsis/pneumonia	46.2	1,336	58.3
Labor and delivery management	23.8	1,215	53.0
Neonatal resuscitation	29.9	332	14.5
Antibiotics for pPRoM^a^	46.2	162	7.1
Clean birth practices	23.8	147	6.4
Immediate assessment and stimulation	23.8	100	4.4
Clean postnatal practices	9.8	93	4.1
Syphilis detection and treatment	23.3	5	0.2
Thermal care	−16.3	−157	−6.9
Injectable antibiotics	−16.3	−259	−11.3
Promotion of breastfeeding	−18.4	−683	−29.8
Total		4,967	100.0

^a^ *pPRoM* preterm premature rupture of membranes

## Discussion

Results of our study quantified the missed opportunities of child survival in Peru if effective interventions don’t reach every mother and child, and if coverage inequality due to household income and location were not addressed. Although currently Peru ranks second among the 75 low and middle income Countdown to 2015 countries in reducing neonatal deaths [14], our analyses showed that further reduction of neonatal deaths is possible through facility-based interventions for small and sick babies, and other facility-based interventions during child birth, among other proven interventions to improve child health.

In the first scenario, interventions with low baseline coverage that are highly effective against high burden diseases would emerge as the missed opportunities in Peru if there is near universal health coverage. Although this scenario did not take into account the feasibility of scale-up of each intervention to cover 90 % of those that needs it, it showed the relative impact of each intervention. Access to full supportive care for prematurity and sepsis/pneumonia, and access to BEmOC and CEmOC level labor and delivery management ranked as the top most impactful interventions. These interventions are health facility-based and are very effective at averting neonatal, maternal deaths and stillbirths [15–19]. Many factors affect priority setting in countries, such as funding availability or societal preferences. Results from this scenario can make a compelling case of using numbers of lives saved as the normative factor to prioritize scale-up of evidence-based, effective health interventions. Policy makers can use the ranking of interventions by lives saved to inform intervention prioritization discussions.

The second and third scenario explored the coverage difference between sub-populations, and the impact if these coverage differences are eliminated. As seen in Tables 1, 3, 4 and 6, coverage estimates between the national and richest population, and between populations in the urban and rural area can differ up to 50 %. The interventions that differ most in their coverage, full supportive care for prematurity and sepsis/pneumonia, are interventions that needed to be delivered in health facilities of CEmOC or BEmOC level. Full supportive care for prematurity include treatment with continuous positive airway pressure (CPAP) and surfactant for preterm babies in health facilities, and other secondary level care including kangaroo mother care and thermal care [15, 16, 20]. Full supportive care for sepsis/pneumonia is also facility-based and include oxygen, IV fluids, IV antibiotics, blood transfusion, phototherapy, etc. as needed, and other secondary level care such as oral and injectable antibiotics [16]. These interventions are very effective against neonatal sepsis, pneumonia, and prematurity, which account for 26 % of the under-5 disease burden (Additional file 1: Figure S1). Neonatal deaths account for half of under-5 deaths in the country, and access to full supportive care at the level of the richest or the urban population can prevent close to 80 % of all under-5 deaths prevented.

Not all interventions coverages are lower in the disadvantaged population. Of interest, breastfeeding prevalence is lower in the richest quintile compared to the national average, and is also lower in urban areas compared to rural areas in Peru. This was also observed in other low and middle income countries, and is concerning as some fear that those that are poor or living in rural areas will follow behaviors of their richer, urban counterparts, and discontinue breastfeeding practices for breast milk substitutes as their income grow [21].

Peru has to continue to address the remaining inequalities in household wealth distribution, poverty, and access to health services, especially in the Amazon and the Andean rural areas [1]. According to our analyses, focusing on eliminating inequalities can reduce Peru’s neonatal mortality rate by 40 % (Table 2). This is almost comparable to the 51 % of neonatal mortality reduction Peru has achieved between 2000 to 2013 [1]. In this period, Peru has achieved remarkable success in sustained economic growth, in reduction of poverty and in scaling up anti-poverty interventions such as the conditional cash transfer program JUNTOS. Coverage of health interventions among the poor in particular went up as cash transfer is conditional on utilization of maternal and child health services [1, 22, 23]. The introduction of new vaccines in Peru is another example of the adoption of a pro-poor approach to ensure a more equitable distribution of coverage among socioeconomic groups. Introduction of the *Haemohylus influenzae* type B (Hib), pneumococcal and rotavirus vaccines were first offered to the poorest segment of Peru, and once a high coverage has been achieved by the poorest areas, then the vaccines were introduced to the rest of the country [24, 25]. Due to the pro-poor approach, coverage difference of Hib vaccine between the poor and the rich, and in rural and urban area is at 4.9 and 0.8 % respectively, which is low compared to other interventions (Tables 1 and 4). Policy makers should consider applying this pro-poor approach to ensure the poor and rural population have access to high quality, facility-based care to further reduce neonatal deaths.

Health system strengthening has been recognized as another key to continue improving child health in Peru. In order to further reduce neonatal mortality by focusing on facility-based interventions as suggested by the three scenarios, availability and readiness of health systems building blocks such as human resources and health facilities need to be addressed. Key health system strengthening components to reduce neonatal mortality include improving the skills of existing health workers to provide quality care at birth and for premature and ill newborn, and increasing the health workforce especially in poor and rural areas with incentives [26]. According to the General Directorate of Public Budget of the Ministry of Economics and Finance of Peru, there has been increasing provision of hospitals, such as neonatal and pediatric intensive care units [27], and increased incentives to attract and retain qualified health professionals to work in rural areas [28–30]. These initiatives will help Peru reduce coverage disparities and further improve child survival among those that are poor and in areas that are hard to reach.

Neonatal causes of death (48 %) is the largest contributor to under-five mortality burden in Peru (Additional file 1: Figure S1), however, one cannot ignore the second largest contributor, other causes of death (28 %). These include deaths due to childhood cancers, congenital abnormalities, and from preterm birth complications, but after 28 days. Due to a lack of interventions with published efficacy on reducing these deaths from other causes, there are no interventions in LiST to model to reduce these deaths. This proportion of the disease burden will only get bigger as Peru goes through the epidemiologic transition, reducing its burden of infectious diseases while increasing its burden on non-infectious diseases. Further research is needed to further classify these other causes of death, and to identify effective interventions in reducing these causes of death. These results align with the identified remaining challenge of Peru’s health system to handle more complex causes of maternal, newborn and child deaths, by better managing pregnancy complications and providing neonatal intensive care [1].

Compared to fellow countries that have achieved substantial progress to improve child health, such as Bangladesh, Malawi, Niger, and Tanzania, similar to Peru, existing efforts to ensure equitable coverage of proven preventive, treatment, and nutritional interventions have been credited as one of the common factors that contributed to the progress made thus far on child survival [31–34]. Compared to these countries, one of the reasons why Peru was able to achieve a lower mortality rate might be due to the fact that the average rate of coverage increases among the poor and in rural areas were actually higher than that of the national average [1]. Common remaining challenges for these health systems include further reducing neonatal mortality through closing the gaps in coverage, intentionally targeting interventions to women and newborns in rural areas, and a new focus to provide high-impact packages of intensive newborn care to all small and sick babies [31–34].

There are limitations to the strength of outputs from these scenarios. Modelling mortality impact relies heavily on availability of data that are measured and reported correctly. Validity of results from the scenarios were also dependent on coverage data availability disaggregated by wealth quintiles and by urban–rural residence. Only coverage of key interventions that were measured and reported in the DHS (listed in Additional file 1: Tables S1 and S3) were available for modelling. Assumptions had to be made for coverage of other interventions that had published evidence in preventing deaths. For example, coverage of interventions delivered during childbirth such as access to BEmOC and CEmOC level labor and delivery management are not typically measured and reported. Coverage of skilled birth attendance and health facility delivery, which are typically measured and reported, were hence used as coverage proxies for those interventions. Nutritional interventions that cut mortality through reducing stunting and wasting, such as zinc supplementation and therapeutic feeding, are also not typically measured and reported in surveys. We used the change in stunting and wasting distributions between sub-populations, which are reported in surveys, to project its impact on reducing mortality.

Quality of the health interventions received directly impact the effectiveness of the intervention in preventing deaths. Coverage estimates published in large household surveys capture the use of and need for health interventions, and assume that the health interventions received were delivered under optimal quality. However, as interventions are unlikely to be delivered under optimal conditions, the effective coverage, one that also takes into account the quality component of the health intervention received, will likely be lower than coverage estimates currently obtained from large household surveys [35]. As a result, if the lower effective coverages were used in our analyses, one would expect to see even larger numbers of additional deaths prevented due to near universal health coverage.

There are many positive impacts stemming from health interventions reaching every mother and every child in an equitable fashion. We have only quantified the mortality impacts, however, there are other impacts associated with increasing coverage of these interventions, such as reduced risks for mortality, decreased incidence of diseases, impacts on children beyond age five, and also impacts beyond improving health. Further studies can be done to quantify these impacts beyond mortality reduction to provide a comprehensive profile of the significance of investing in health of women and children. Although our study quantified the missed opportunities of child survival in Peru, decision makers must re-examine the identified factors that have led to Peru’s success in reducing neonatal and child mortality in the past decade, and build on these knowledges to pave the way forward for universal and equitable coverage of these effective interventions.

## Conclusions

Building on the momentum of success that Peru has achieved in the past decade with child mortality reduction, results from our analyses show that more work can be and has to be done to reduce mortality, particularly neonatal mortality. The bulk of the missed opportunities in Peru lie in neonatal prematurity and sepsis deaths, and can be mitigated through scaling up effective health facility-based case management for all small and sick newborns, and through eliminating coverage gaps due to household wealth and location. Next steps will involve identifying pockets of population that are poor and reside in rural areas, understanding factors that contribute to their low utilization of facility-based interventions, and directing resources for intervention scale-up. Evidence from this study should inform further efforts and direction to improve neonatal and child health in Peru, and contribute to reduce preventable newborn and child health to attain the Sustainable Development Goals (SDGs) by 2030.

## Additional file

Additional file 1: Table 1. Coverage estimates for baseline 2012 Peru’s national coverage and target 2013 Peru’s richest coverage. All coverage estimates were published in DHS 2012. **Table 2**. Baseline indicators for national Peru. **Figure 1**. Mortality burden in children under five years of age in national Peru in 2012. **Table 3**. Coverage estimates for baseline 2012 Peru’s rural coverage and target 2013 Peru’s urban coverage. All coverage estimates were published in DHS 2012. **Table 4**. Baseline indicator for rural Peru. **Figure 2**. Mortality burden in children under five years of age in rural Peru in 2012. Source: Projected by LiST. **Figure 3**. Number of maternal deaths prevented by intervention in national Peru, 2013-2017, scenario ‘Universal coverage’. **Figure 4**. Number of stillbirths prevented by intervention in national Peru, 2013-2017, scenario ‘Universal coverage’. **Figure 5**. Number of neonatal deaths prevented by intervention in national Peru, 2013-2017, scenario ‘Universal coverage’. **Figure 6**. Number of 1-59 months child deaths prevented by intervention in national Peru, 2013-2017, scenario ‘Universal coverage’. (DOCX 48 kb)

## Abbreviations

BEmOC: Basic emergency obstetric care
CEmOC: Comprehensive Emergency Obstetric Care
CHERG: Child Health Epidemiology Reference Group
CPAP: Continuous positive airway pressure
DHS: Demographic Health Survey
Hib: Haemophilus Influenzae Type B
LiST: Lives Saved Tool
pPRoM: Preterm premature rupture of membranes

## References

[1] Huicho L, Segura ER, Huayanay-Espinoza CA, de Guzman JN, Restrepo-Méndez MC, Tam Y, Barros AJD, Victora CG (2016). Child health and nutrition in Peru within an antipoverty political agenda: a countdown to 2015 country case study. Lancet Glob Health.

[2] Jennifer R, Cesar V, Jennifer B: A Decade of Tracking Progress for Maternal, Newborn and Child Survival. The Countdown 2015 Report. In.: UNICEF & WHO; 2015.

[3] Walker N, Tam Y, Friberg IK (2013). Overview of the Lives Saved Tool (LiST). BMC Public Health.

[4] Walker N, Fischer-Walker C, Bryce J, Bahl R, Cousens S (2010). Effects CRGoI: standards for CHERG reviews of intervention effects on child survival. Int J Epidemiol.

[5] Victora CG (2010). LiST: using epidemiology to guide child survival policymaking and programming. Int J Epidemiol.

[6] Stover J, McKinnon R, Winfrey B (2010). Spectrum: a model platform for linking maternal and child survival interventions with AIDS, family planning and demographic projections. Int J Epidemiol.

[7] Peru: Continuous DHS, 2012 [http://dhsprogram.com/what-we-do/survey/survey-display-434.cfm]. Accessed July 2015.

[8] Microdatos. Base de Datos. [http://iinei.inei.gob.pe/microdatos/]. Accessed July 2015.

[9] Liu L, Oza S, Hogan D, Perin J, Rudan I, Lawn JE, Cousens S, Mathers C, Black RE (2015). Global, regional, and national causes of child mortality in 2000–13, with projections to inform post-2015 priorities: an updated systematic analysis. Lancet.

[10] World Population Prospects. The 2012 Revision [http://esa.un.org/unpd/wpp/Publications/Files/WPP2012_HIGHLIGHTS.pdf]. Accessed July 2015.

[11] Child Mortality Estimates-Peru [http://www.childmortality.org]. Accessed July 2015.

[12] Amouzou A, Richard SA, Friberg IK, Bryce J, Baqui AH, El Arifeen S, Walker N (2010). How well does LiST capture mortality by wealth quintile? A comparison of measured versus modelled mortality rates among children under-five in Bangladesh. Int J Epidemiol.

[13] WHO vaccine-preventable diseases: monitoring system 2016 global summary - Time Series: DTP3 [http://apps.who.int/immunization_monitoring/globalsummary/timeseries/tscoveragedtp3.html]. Accessed July 2015.

[14] Geoghegan T (2013). State of the World’s Mothers 2013.

[15] Zaidi AK, Ganatra HA, Syed S, Cousens S, Lee AC, Black R, Bhutta ZA, Lawn JE (2011). Effect of case management on neonatal mortality due to sepsis and pneumonia. BMC Public Health.

[16] Bhutta ZA, Das JK, Bahl R, Lawn JE, Salam RA, Paul VK, Sankar MJ, Blencowe H, Rizvi A, Chou VB (2014). Can available interventions end preventable deaths in mothers, newborn babies, and stillbirths, and at what cost?. Lancet.

[17] Lee AC, Cousens S, Darmstadt GL, Blencowe H, Pattinson R, Moran NF, Hofmeyr GJ, Haws RA, Bhutta SZ, Lawn JE (2011). Care during labor and birth for the prevention of intrapartum-related neonatal deaths: a systematic review and Delphi estimation of mortality effect. BMC Public Health.

[18] Yakoob MY, Ali MA, Ali MU, Imdad A, Lawn JE, Van Den Broek N, Bhutta ZA (2011). The effect of providing skilled birth attendance and emergency obstetric care in preventing stillbirths. BMC Public Health.

[19] Pollard SL, Mathai M, Walker N (2013). Estimating the impact of interventions on cause-specific maternal mortality: a Delphi approach. BMC Public Health.

[20] Lawn JE, Davidge R, Paul VK, von Xylander S, de Graft Johnson J, Costello A, Kinney MV, Segre J, Molyneux L (2013). Born too soon: care for the preterm baby. Reprod Health.

[21] Victora CG, Bahl R, Barros AJ, Franca GV, Horton S, Krasevec J, Murch S, Sankar MJ, Walker N, Rollins NC (2016). Breastfeeding in the 21st century: epidemiology, mechanisms, and lifelong effect. Lancet.

[22] Peru Overview [http://www.worldbank.org/en/country/peru/overview]. Accessed Aug 2015.

[23] Inchauste G, Olivieri S, Saavedra J, Winkler H (2012). What Is Behind The Decline in Poverty Since 2000? Evidence from Bangladesh, Peru, and Thailand.

[24] Considerations for Incorporating Health Equity into Project Designs: A Guide for Community-Oriented Maternal, Neonatal, and Child Health Projects, [http://www.mchip.net/sites/default/files/Equity%20guidance_090111_formatted_final.pdf]. Accessed Aug 2015.

[25] Tercer Informe. Intervención Pública Evaluada: Servicio de Vacunación. [http://www.mef.gob.pe/contenidos/presu_publ/ppr/eval_indep/2010_informe_final_VACUNACIONES.pdf]

[26] Dickson KE, Simen-Kapeu A, Kinney MV, Huicho L, Vesel L, Lackritz E, de Graft JJ, von Xylander S, Rafique N, Sylla M (2014). Every newborn: health-systems bottlenecks and strategies to accelerate scale-up in countries. Lancet.

[27] Progreso en los resultados del Programa Estratégico Salud Materno Neonatal [http://www.mef.gob.pe/contenidos/presu_publ/ppr/PPR_salud_materno.pdf]. Accessed Aug 2015.

[28] Huicho L, Miranda JJ, Diez-Canseco F, Lema C, Lescano AG, Lagarde M, Blaauw D (2012). Job preferences of nurses and midwives for taking up a rural job in Peru: a discrete choice experiment. PLoS One.

[29] Miranda JJ, Diez-Canseco F, Lema C, Lescano AG, Lagarde M, Blaauw D, Huicho L (2012). Stated preferences of doctors for choosing a job in rural areas of Peru: a discrete choice experiment. PLoS One.

[30] Huicho L, Dieleman M, Campbell J, Codjia L, Balabanova D, Dussault G, Dolea C (2010). Increasing access to health workers in underserved areas: a conceptual framework for measuring results. Bull World Health Organ.

[31] El Arifeen S, Hill K, Ahsan KZ, Jamil K, Nahar Q, Streatfield PK (2014). Maternal mortality in Bangladesh: a countdown to 2015 country case study. Lancet.

[32] Kanyuka M, Ndawala J, Mleme T, Chisesa L, Makwemba M, Amouzou A, Borghi J, Daire J, Ferrabee R, Hazel E (2016). Malawi and Millennium Development Goal 4: a countdown to 2015 country case study. Lancet Glob Health.

[33] Amouzou A, Habi O, Bensaid K (2012). Reduction in child mortality in Niger: a countdown to 2015 country case study. Lancet.

[34] Afnan-Holmes H, Magoma M, John T, Levira F, Msemo G, Armstrong CE, Martinez-Alvarez M, Kerber K, Kihinga C, Makuwani A (2015). Tanzania’s countdown to 2015: an analysis of two decades of progress and gaps for reproductive, maternal, newborn, and child health, to inform priorities for post-2015. Lancet Glob Health.

[35] Ng M, Fullman N, Dieleman JL, Flaxman AD, Murray CJL, Lim SS. Effective coverage: a metric for monitoring universal health coverage. PLoS Med. 2014;11(9):e1001730. doi:10.1371/journal.pmed.1001730. 10.1371/journal.pmed.1001730 PMC4171091 25243780

