# Supplementary material for: Remaining missed opportunities of child survival in Peru: modelling mortality impact of universal and equitable coverage of proven interventions
Source: BMC Public Health. 2016 Oct 4;16:1048. doi: 10.1186/s12889-016-3668-7 (PMC5050579; doi:10.1186/s12889-016-3668-7)
Supplement: Additional file 1: Table 1. — Coverage estimates for baseline 2012 Peru’s national coverage and target 2013 Peru’s richest coverage. All coverage estimates were published in DHS 2012. Table 2. Baseline indicators for national Peru. Figure 1. Mortality burden in children under five years of age in national Peru in 2012. Table 3. Coverage estimates for baseline 2012 Peru’s rural coverage and target 2013 Peru’s urban coverage. All coverage estimates were published in DHS 2012. Table 4. Baseline indicator for rural Peru. Figure 2. Mortality burden in children under five years of age in rural Peru in 2012. Source: Projected by LiST. Figure 3. Number of maternal deaths prevented by intervention in national Peru, 2013-2017, scenario ‘Universal coverage’. Figure 4. Number of stillbirths prevented by intervention in national Peru, 2013-2017, scenario ‘Universal coverage’. Figure 5. Number of neonatal deaths prevented by intervention in national Peru, 2013-2017, scenario ‘Universal coverage’. Figure 6. Number of 1-59 months child deaths prevented by intervention in national Peru, 2013-2017, scenario ‘Universal coverage’. (DOCX 48 kb) [file 12889_2016_3668_MOESM1_ESM.docx]

Table 1. Coverage estimates for baseline 2012 Peru’s national coverage and target 2013 Peru’s richest coverage. All coverage estimates were published in DHS 2012.

| **Intervention categories and age groups** | **Intervention (%)** | **Definition** | **National coverage (%)** | **Richest coverage (%)** |
| --- | --- | --- | --- | --- |
| **Pregnancy** | | | | |
|  | Antenatal care | Percentage of women attended four or more times during pregnancy by any provider | 94.2 | 99.1 |
|  | TT - Tetanus toxoid vaccination | Percentage of newborns protected against tetanus | 85.0 | 85.0 |
| **Childbirth** | | | | |
|  | Skilled birth attendance | Percentage of live births attended by skilled health personnel | 88.4 | 99.5 |
|  | Health facility delivery | Percent of children born in a health facility. | 84.7 | 98.5 |
| **Breastfeeding** | | | | |
| < 1 month | Exclusive breastfeeding | Percent of children receiving only breastmilk for food (plus medication, vaccines, and vitamins). | 68.9 | 48.0 |
|  | Predominant breastfeeding | Percent of children receiving only breastmilk plus water and/or other non-milk liquids such as juices (plus medication, vaccines, and vitamins). | 4.8 | 0.0 |
|  | Partial breastfeeding | Percent of children receiving breastmilk plus complementary foods and/or milk-based liquids (plus medication, vaccines, and vitamins). | 22.5 | 52.0 |
|  | Not breastfeeding | The percent of children not receiving any breastmilk. | 3.8 | 0.0 |
| 1 to 5 months | Exclusive breastfeeding |  | 67.5 | 49.7 |
|  | Predominant breastfeeding |  | 4.8 | 0.0 |
|  | Partial breastfeeding |  | 25.2 | 38.0 |
|  | Not breastfeeding |  | 2.5 | 12.3 |
| 6 to 11 months | Any breastfeeding | The percent of children still receiving any breastmilk. | 92.0 | 69.4 |
|  | Not breastfeeding | The percent of children not receiving any breastmilk. | 8.0 | 30.6 |
| 12 to 23 months | Any breastfeeding |  | 65.8 | 52.5 |
|  | Not breastfeeding |  | 34.2 | 47.5 |
| **Preventive** | | | | |
|  | Clean postnatal practices | Percentage of babies who received postnatal care within two days of childbirth | 92.3 | 96.9 |
|  | Vitamin A supplementation | Percent of children 6-59 months of age receiving two doses of Vitamin A during the last 12 months. | 4.5 | 1.0 |
|  | Improved water source | Percentage of the population using improved drinking water sources (piped on premises or other improved drinking water sources) | 86.5 | 99.7 |
|  | Water connection in the home | Percent of households with a household connection, including water piped into the home or yard. | 81.8 | 99.7 |
|  | Improved sanitation - Utilization of latrines or toilets | Percentage of the population using improved sanitation facilities | 60.9 | 98.7 |
|  | Hygienic disposal of children’s stools | Percent of children's stools that are disposed of safely and contained. Stools are considered to be contained if: 1) the child always uses a toilet/latrine, 2) the faeces are thrown in the toilet/latrine, or 3) the faeces are buried in the yard. | 51.2 | 45.8 |
|  | ITN/IRS - Ownership of insecticide treated nets (ITN/LLIN) or household protected with indoor residual spraying | Percent of households owning at least one insecticide treated bednet (ITN) or protected by indoor residual spraying (IRS). | 0.0 | 0.0 |
| **Vaccines** | | | | |
|  | DPT | Percentage of children 12 to 23 months who received three doses of diphtheria/pertussis/tetanus vaccine | 83.7 | 88.6 |
|  | *H. influenzae* b | Percentage of children 12 to 23 months who received three doses of *Haemophilus influenzae* type B vaccine | 83.9 | 88.8 |
|  | Pneumococcal | Percent of children 12-23 months who have received 3 doses of Pneumococcal vaccine. | 89.0 | 89.0 |
|  | Rotavirus | Percent of children 12-23 months who have received 2 or 3 doses of Rotavirus vaccine (according to manufacturer’s schedule). | 91.0 | 91.0 |
|  | Measles | Percentage of children 12 to 23 months who have received 1 dose of measles vaccine | 89.5 | 89.1 |
| **Curative** | | | | |
|  | ORS - oral rehydration solution | Percentage of children ages 0–59 months with diarrhoea receiving oral rehydration salts | 30.9 | 32.8 |
|  | Oral antibiotics for pneumonia | Percentage of children ages 0–59 months with symptoms of pneumonia taken to an appropriate health provider | 67.2 | 68.4 |
|  | Antimalarials - Artemesinin compounds for malaria | Percent of children treated within 48 hours of the onset of fever in malaria-endemic areas with an artemesinin-containing compound (artemisinin-based combination therapy, or ACT). | 0.0 | 0.0 |

Table 2. Baseline indicators for national Peru.

| **Baseline indicator for national Peru** | **2012 Estimate** | **Source** |
| --- | --- | --- |
| Total population | 29,373,890 | UN Population Division World Population Prospects |
| Total fertility rate | 2.43% |  |
| Neonatal mortality rate | 9.4% | UN Inter-agency Group for Child Mortality Estimation (IGME) |
| Under-five mortality rate | 19.1% |  |

Figure 1. Mortality burden in children under five years of age in national Peru in 2012.

Note: There are no neonatal diarrhoea, measles or malaria death in Peru. “Other” category consists of deaths due to childhood cancers, congenital abnormalities, and from preterm birth complications, but after 28 days.

Table 3. Coverage estimates for baseline 2012 Peru’s rural coverage and target 2013 Peru’s urban coverage. All coverage estimates were published in DHS 2012.

| **Intervention categories and age groups** | **Intervention (%)** | **Definition** | **Rural coverage (%)** | **Urban coverage (%)** |
| --- | --- | --- | --- | --- |
| **Periconceptual** | | | | |
|  | Contraceptive use | Proportion of women currently married or in union aged 15–49 that are using (or whose partner is using) a contraceptive method (either modern or traditional) | 74.8 | 75.8 |
| **Pregnancy** | | | | |
|  | Antenatal care | Percentage of women attended four or more times during pregnancy by any provider | 90.7 | 95.9 |
|  | TT - Tetanus toxoid vaccination | Percentage of newborns protected against tetanus | 85.0 | 85.0 |
|  | IPTp - Pregnant women protected via intermittent preventive treatment of malaria during pregnancy or by sleeping under an ITN | Percentage of women who received intermittent preventive treatment for malaria during their last pregnancy | 0.0 | 0.0 |
| **Childbirth** | | | | |
|  | Skilled birth attendance | Percentage of live births attended by skilled health personnel | 72.5 | 96.3 |
|  | Health facility delivery | Percent of children born in a health facility. | 65.1 | 95.0 |
| **Breastfeeding** | | | | |
| < 1 month | Exclusive breastfeeding | Percent of children receiving only breastmilk for food (plus medication, vaccines, and vitamins). | 65.1 | 70.0 |
|  | Predominant breastfeeding | Percent of children receiving only breastmilk plus water and/or other non-milk liquids such as juices (plus medication, vaccines, and vitamins). | 17.1 | 1.4 |
|  | Partial breastfeeding | Percent of children receiving breastmilk plus complementary foods and/or milk-based liquids (plus medication, vaccines, and vitamins). | 17.8 | 23.7 |
|  | Not breastfeeding | The percent of children not receiving any breastmilk. | 0.0 | 4.9 |
| 1 to 5 months | Exclusive breastfeeding |  | 79.3 | 60.9 |
|  | Predominant breastfeeding |  | 3.0 | 5.8 |
|  | Partial breastfeeding |  | 16.6 | 29.9 |
|  | Not breastfeeding |  | 1.1 | 3.4 |
| 6 to 11 months | Any breastfeeding | The percent of children still receiving any breastmilk. | 99.4 | 89.0 |
|  | Not breastfeeding | The percent of children not receiving any breastmilk. | 0.6 | 11.0 |
| 12 to 23 months | Any breastfeeding |  | 73.9 | 61.3 |
|  | Not breastfeeding |  | 26.1 | 38.7 |
| **Preventive** | | | | |
|  | Clean postnatal practices | Percentage of babies who received postnatal care within two days of childbirth | 85.8 | 95.6 |
|  | Vitamin A supplementation | Percent of children 6-59 months of age receiving two doses of Vitamin A during the last 12 months. | 8.4 | 2.5 |
|  | Improved water source | Percentage of the population using improved drinking water sources (piped on premises or other improved drinking water sources) | 71.6 | 93.1 |
|  | Water connection in the home | Percent of households with a household connection, including water piped into the home or yard. | 64.2 | 89.5 |
|  | Improved sanitation - Utilization of latrines or toilets | Percentage of the population using improved sanitation facilities | 28.9 | 75.0 |
|  | Hygienic disposal of children's stools | Percent of children's stools that are disposed of safely and contained. Stools are considered to be contained if: 1) the child always uses a toilet/latrine, 2) the faeces are thrown in the toilet/latrine, or 3) the faeces are buried in the yard. | 49.8 | 51.9 |
|  | ITN/IRS - Ownership of insecticide treated nets (ITN/LLIN) or household protected with indoor residual spraying | Percent of households owning at least one insecticide treated bednet (ITN) or protected by indoor residual spraying (IRS). | 0.0 | 0.0 |
| **Vaccines** | | | | |
|  | DPT | Percentage of children 12 to 23 months who received three doses of diphtheria/pertussis/tetanus vaccine | 82.8 | 84.2 |
|  | *H. influenzae b* | Percentage of children 12 to 23 months who received three doses of *Haemophilus influenzae* type B vaccine | 83.4 | 84.2 |
|  | Pneumococcal | Percent of children 12-23 months who have received 3 doses of Pneumococcal vaccine. | 89.0 | 89.0 |
|  | Rotavirus | Percent of children 12-23 months who have received 2 or 3 doses of Rotavirus vaccine (according to manufacturer’s schedule). | 91.0 | 91.0 |
|  | Measles | Percentage of children 12 to 23 months who have received 1 dose of measles vaccine | 90.5 | 89.0 |
| **Curative** | | | | |
|  | ORS - oral rehydration solution | Percentage of children ages 0–59 months with diarrhoea receiving oral rehydration salts | 23.2 | 35.2 |
|  | Oral antibiotics for pneumonia | Percentage of children ages 0–59 months with symptoms of pneumonia taken to an appropriate health provider | 65.8 | 67.8 |
|  | Antimalarials - Artemesinin compounds for malaria | Percent of children treated within 48 hours of the onset of fever in malaria-endemic areas with an artemesinin-containing compound (artemisinin-based combination therapy, or ACT). | 0.0 | 0.0 |

Table 4. Baseline indicator for rural Peru.

| **Baseline indicator** | **Estimate** | **Source** |
| --- | --- | --- |
| Total population | 6,785,369 | Estimated as 23.1% of the national population by World Bank |
| Total fertility rate | 5.38% | DHS 2012 |
| Neonatal mortality rate | 11.1% | Projected by LiST |
| Under-five mortality rate | 22.3% | Projected by LiST |

Figure 2. Mortality burden in children under five years of age in rural Peru in 2012. Source: Projected by LiST.

Note: There are no neonatal diarrhea, measles, or malaria death in rural Peru. “Other” category consists of deaths due to childhood cancers, congenital abnormalities, and from preterm birth complications, but after 28 days.

Figure 3. Number of maternal deaths prevented by intervention in national Peru, 2013-2017, scenario ‘Universal coverage’

Figure 4. Number of stillbirths prevented by intervention in national Peru, 2013-2017, scenario ‘Universal coverage’

Figure 5. Number of neonatal deaths prevented by intervention in national Peru, 2013-2017, scenario ‘Universal coverage’

Figure 6. Number of 1-59 months child deaths prevented by intervention in national Peru, 2013-2017, scenario ‘Universal coverage’
